# Octanoylated Ghrelin Inhibits the Activation of the Palmitic Acid-Induced TLR4/NF-**κ**B Signaling Pathway in THP-1 Macrophages

**DOI:** 10.5402/2012/237613

**Published:** 2012-11-26

**Authors:** S. P. Liu, X. Y. Li, Z. Li, L. N. He, Y. Xiao, K. Yan, Z. G. Zhou

**Affiliations:** Diabetes Center, 2nd Xiangya Hospital, Institute of Metabolism and Endocrinology, Key Laboratory of Diabetes Immunology of Ministry of Education, Central South University, 139 Renmin-Zhong Road, Hunan, Changsha 410011, China

## Abstract

To investigate the effect of acylated ghrelin on the activation of TLR4/NF-**κ**B signaling pathway induced by palmitic acid in human monocyte-derived (THP-1) macrophages, THP-1 macrophages were cultured for 12 h by palmitic acid with various concentrations. The THP-1 macrophages was pretreated by acylated ghrelin at different doses for 4 h before cultivated by palmitic acid (200 **μ**mol/L) for 12 h. We observed the level of TLR4, NF-**κ**B p65 phosphorylation in THP-1 macrophages and TNF-**α**, IL-1**β** in culture supernatant. TLR4 mRNA was measured by real-time PCR. TLR4 protein and NF-**κ**B p65 phosphorylation was measured by western blotting. The expression of TNF-**α** and IL-1**β** was detected by ELISA. Compared to the THP-1 macrophages without palmitic acid, the level of TLR4 mRNA protein and NF-**κ**B p65 phosphorylation and the expression of TNF-**α** and IL-1**β** increased after treatment by palmitic acid in a dose-dependent fashion (*P* < 0.05). Compared to the THP-1 macrophages with palmitic acid (200 **μ**mol/L), the level of the pervious substances decreased after preadministration by acylated ghrelin in a dose-dependent fashion. So, we make a conclusion that acylated ghrelin can regulate the activation of TLR4/NF-**κ**B signaling pathway and inhibit the release of inflammatory cytokines in THP-1 macrophages which are stimulated by palmitic acid in a dose-dependent fashion.

## 1. Introduction

Obesity is a chronic disease that is associated with low-grade inflammation [1–3]. Obesity can induce changes in metabolism and gene expression in adipocytes, thereby enhancing lipolysis and releasing proinflammatory free fatty acids (FFAs) and cytokines (such as monocyte chemotactic protein-1 (MCP-1) and tumor necrosis factor-alpha (TNF-*α*)), which are able to recruit and activate macrophages to produce high levels of proinflammatory mediators (such as TNF-*α*, interleukin-1 beta (IL-1*β*), and resistin). These proinflammatory factors can promote an insulin-resistant state in adipocytes and form a positive feedback loop. The establishment of this positive feedback pathway can further amplify the inflammatory response and insulin resistance [4]. Obesity is related to metabolic inflammation, which is not only the basic pathophysiological mechanism for insulin resistance but is also critical in the occurrence of obesity-related vascular complications. Obesity has been shown to be associated with the activation of the innate immunity pathway and inflammatory response, as well as an impaired insulin signaling pathway and insulin resistance [5–10].

Toll-like receptor 4 (TLR4) could serve as a bridge that links innate immunity, lipid metabolism, and insulin resistance [11, 12]. Patients with overnutrition and obesity often have elevated levels of FFAs in their circulatory system because large quantities of saturated fatty acids are released by lipolysis from hypertrophied adipocytes due to the macrophages' activities. These fatty acids serve as a natural endogenous ligand of TLR4, thereby activating downstream targets, such as the proinflammatory transcription factor nuclear factor-kappa B (NF-*κ*B), to mediate an inflammatory response and insulin resistance. In addition, TLR4 can also activate the transformation from monocytes to macrophages, thereby forming foam cells that lead to the formation of atherosclerotic plaques. In these previously mentioned processes, the NF-*κ*B pathway plays an important role in the TLR4-mediated regulation of immunity [4, 9, 11–15].

Palmitic acid, also known as alkyl [16] acid, is a saturated fatty acid. Palmitic acid acts as a natural dietary ligand for the activation of TLR4 signal transduction, which ultimately leads to the activation of NF-*κ*B and activator protein-1 in macrophages and adipocytes and promotes the release of proinflammatory cytokines and chemokines. Ghrelin is a gastrointestinal peptide hormone that is secreted by gastric X/A-like endocrine cells. Octanoylated ghrelin is the activated form of ghrelin, which can bind with its endogenous ligand, the growth hormone secretagogue receptor (GHSR), and has multiple biological functions, including potential therapeutic effects on the cardiovascular system. In particular, ghrelin has been shown to have significant anti-inflammatory effects in both animal experiments and *in vitro *studies [17–22]. Therefore, we explored the effects of octanoylated ghrelin on the activation of the TLR4 signaling pathway and the secretion of inflammatory factors from palmitic acid-induced human monocyte-derived (THP-1) macrophages.

## 2. Materials and Methods

### 2.1. Cell Culture and Treatment

The THP-1 monocytic cell line (Shanghai Institute of Biological Sciences) was used in this study. THP-1 monocytes were cultured in complete culture medium containing 100 nM phorbol-12-myristate-13-acetate (Sigma, USA) for 72 hours to induce the differentiation of THP-1 macrophages. After the medium was discarded, the macrophages were cultured in serum-free medium for 12 hours. Two separate experiments were then performed. In the first experiment, THP-1 macrophages were incubated with different concentrations (0 *μ*mol/L, 100 *μ*mol/L, 200 *μ*mol/L, and 500 *μ*mol/L) of palmitic acid and 10 ng/mL lipopolysaccharide (LPS) for 12 hours. The second experiment included the following five groups: a control group (medium containing fetal bovine serum); a 200 *μ*mol/L palmitic acid-incubated group; a 1 nmol/L octanoylated ghrelin (AnaSpec) + 200 *μ*mol/L palmitic acid intervention group; a 10 nmol/L octanoylated ghrelin + 200 *μ*mol/L palmitic acid intervention group; a 100 nmol/L octanoylated ghrelin + 200 *μ*mol/L palmitic acid intervention group. Different concentrations of octanoylated ghrelin were added to THP-1 macrophages in each of the groups, and the cells were incubated for 4 hours. Palmitic acid (200 *μ*mol/L) was then added followed by 12 hours of incubation. The cells from all of the groups were collected for protein and mRNA extractions. The cell culture supernatants were also collected and stored in at −70°C prior to TNF-*α* and IL-1*β* analyses. 

### 2.2. RT-PCR

 Total RNA was extracted from the THP-1 cells using TRIzol after the intervention. A UV protein and nucleic acid analyzer was used to determine the RNA concentration and purity, which was based on an OD260/OD280 ratio between 1.8 and 2.0. The 18S gene was used as an internal reference gene. The primers for human TLR4 included the sense primer 5′-AAGCCGAAAGGTGATTGTTG-3′ and the antisense primer 5′-CTGAGCAGGGTCTTCTCCAC-3′. The primers for the internal reference gene (18S) included the sense primer 5′-AGTCCCTGCCCTTTGTACACA-3′ and the antisense primer 5′-CGATCCGAGGGCCTCACTA-3′. A One Step SYBR PrimeScript RT-PCR Kit II (Takara) system was used according to the manufacturer's instructions. The amplification and melting curves were used to calculate the Ct value of the TLR4 gene and the difference between the Ct values (ΔCt) of the TLR4 and 18S genes. In addition, the equation 2^−ΔΔCt^ was used to determine the relative amount of TLR4 mRNA.

### 2.3. Western Blotting

After the THP-1 macrophages were collected, they were washed twice with PBS. The cells were then lysed in a radioimmunoprecipitation assay buffer and phenylmethanesulfonyl fluoride mixture at a 98 : 2 ratio to extract the proteins. The protein levels in each group were adjusted to the same level based on a bicinchoninic acid protein assay kit. For each group, 40 *μ*g of the protein sample was mixed with sodium dodecyl sulfate (SDS) gel-loading buffer (20% w/v) and was boiled at 98°C for five minutes. *β*-actin was used as an internal reference. Following SDS-polyacrylamide gel electrophoresis (SDS-PAGE) and protein transfer to polyvinylidene difluoride (PVDF) membranes, the membranes were blocked in a TBST (Tris-buffered saline/TWEEN-20) solution containing 10% nonfat dry milk for 1 hour. The primary antibodies for all of the proteins were proportionally diluted into working solutions with antibody diluents. The PVDF membranes were incubated in the primary antibody solution overnight at 4°C. Subsequently, the primary antibody solution was discarded. The membranes were rinsed three times in TBST for 15 minutes on a shaking table. The secondary antibody working solutions were prepared using the secondary antibody diluted in 5% nonfat milk at a ratio of 1 : 3000. The PVDF membranes were incubated in the secondary antibody solution at room temperature for 1 hour and were subsequently rinsed three times (15 minutes each rinse). Enhanced chemiluminescence reagents were used for staining. The films were scanned thereafter and stored using Image Lab software. Image J software was used to determine the gray value of each protein band. The gray value ratios of the TLR4 and phosphorylated NF-*κ*B bands over the internal reference *β*-actin band were used to quantify the expression of TLR4 and phosphorylated NF-*κ*B.

### 2.4. Measurement of IL-1*β* and TNF-*α* by ELISA

The cytokine measurements were performed according to the manufacturer's instructions for the ELISA kit (BD Biosciences, USA).

### 2.5. Statistical Analysis

Statistical comparisons were conducted using a one-way analysis of variance (ANOVA) and least significant difference test. 

## 3. Results

**Figure 1:**
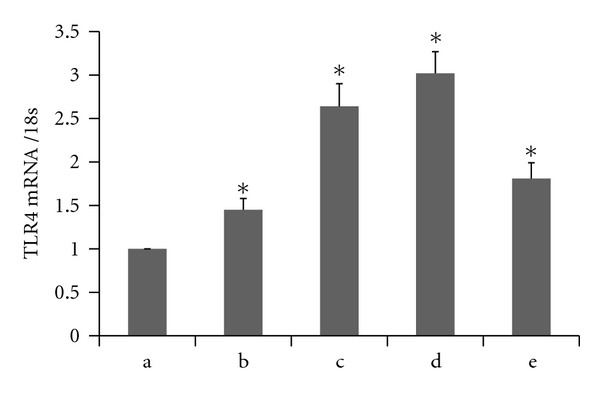
Palmitic acid augments the level of TLR4 mRNA in THP-1 macrophages by real-time RT-PCR analysis. (a) no treatment; (b) palmitic acid 100 *μ*mol/L group; (c) palmitic acid 200 *μ*mol/L group; (d) palmitic acid 500 *μ*mol/L; (e) LPS 10 ng/mL group. Values are expressed as mean ± SEM (*P < 0.05 versus no treatment).

**Figure 2:**
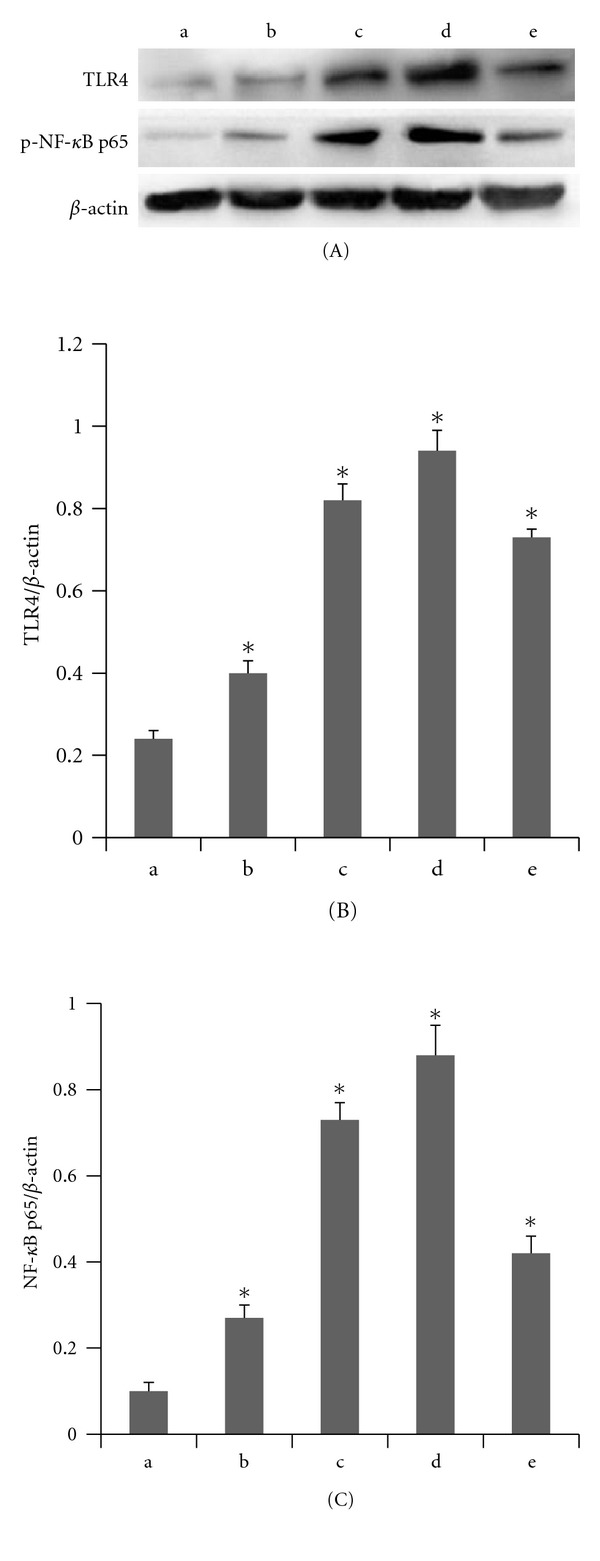
Palmitic acid augmented the level of TLR4 protein and NF-*κ*B p65 phosphorylationprotein in THP-1 macrophages. (a) control; (b) palmitic acid 100 *μ*mol/L group; (c) palmitic acid 200 *μ*mol/L group; (d) palmitic acid 500 *μ*mol/L; (e) LPS 10 ng/mL group. (A) Western blotting electropherogram of TLR4 (96 KD), NF-*κ*B p65 (65 KD), and *β*-actin (42 KD). (B) Palmitic acid augmented the level of TLR4 protein in a dose-dependent in THP-1 macrophages, with values expressed as mean ± SEM (∗*P* < 0.05 versus control). (C) Palmitic acid augmented the level of NF-*κ*B p65 phosphorylation protein in a dose-dependent in THP-1 macrophages, with values expressed as mean ± SEM (*P < 0.05 versus control).

**Figure 3:**
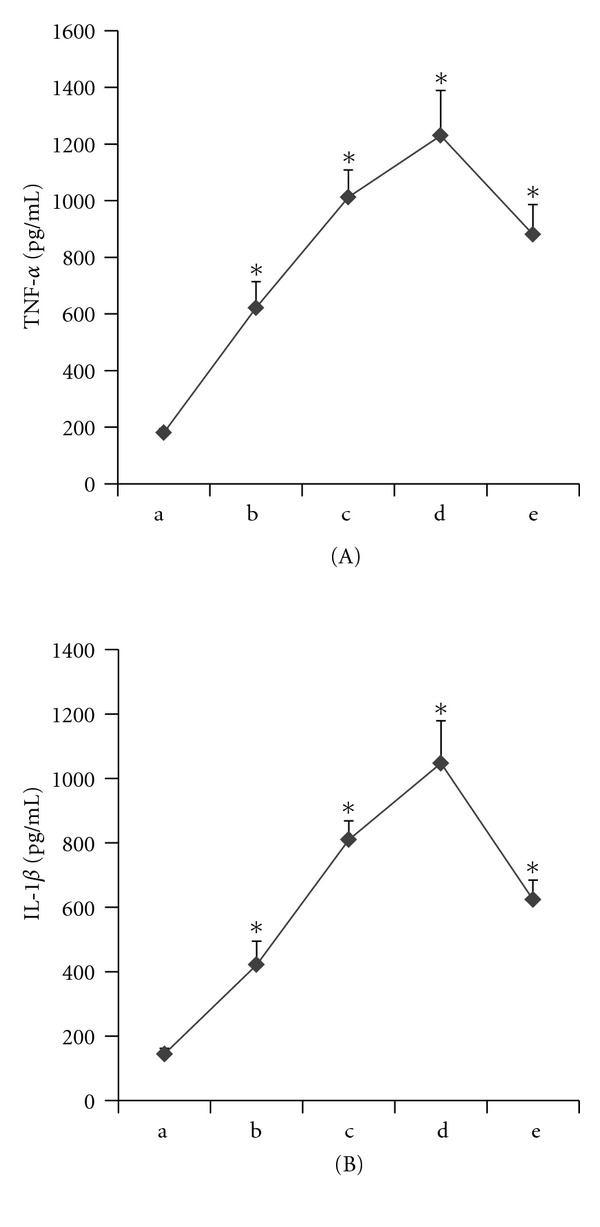
Palmitic acid augmented the expression of IL-1*β* and TNF-*α* in THP-1 macrophages. (a) control; (b) palmitic acid 100 *μ*mol/L group; (c) palmitic acid 200 *μ*mol/L group; (d) palmitic acid 500 *μ*mol/L; (e) LPS 10 ng/mL group. (A) Palmitic acid augmented the expression of TNF-*α* in a dose-dependent in THP-1 macrophages, with values expressed as mean ± SEM (*P < 0.05 versus control). (B) Palmitic acid augmented the expression of IL-1*β* in a dose-dependent in THP-1 macrophages, with values expressed as mean ± SEM (*P < 0.05 versus control).

The effects of palmitic acid on TLR4, intracellular NF-*κ*B p65 phosphorylation, and the TNF-*α* and IL-1*β* levels in THP-1 macrophages are shown in Figures 1–3.

In our study, THP-1 macrophages were incubated with different concentrations of palmitic acid (100 *μ*mol/L, 200 *μ*mol/L, and 500 *μ*mol/L) and 10 ng/mL LPS for 12 hours. The RT-PCR results showed that the TLR4 mRNA expression increased with increasing palmitic acid concentrations. These results were all statistically significant (*P* < 0.05) relative to the control group (Figure 1). The western blot analyses showed that palmitic acid increased the TLR4 and phosphorylated NF-*κ*B p65 protein levels in THP-1 macrophages in a dose-dependent manner. These differences were all statistically significant (*P* < 0.05) relative to the control group (Figure 2). The ELISA results showed that palmitic acid increased the secretion of IL-1*β* and TNF-*α* by THP-1 macrophages in a dose-dependent manner. These results were also statistically significant (*P* < 0.05) relative to the control group (Figure 3).

**Figure 4:**
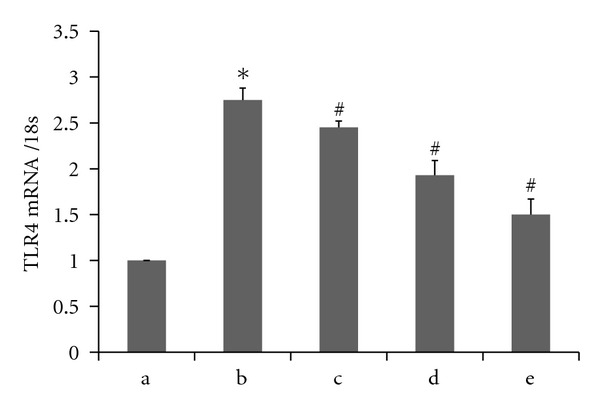
Acylated ghrelin pretreatment decreased palmitic acid-induced level of TLR4 mRNA in THP-1 macrophages by real-time RT-PCR analysis. (a) control; (b) palmitic acid 200 *μ*mol/L group; (c) ghrelin 1 nmol/L+palmitic acid 200 *μ*mol/L group; (d) ghrelin 10 nmol/L+palmitic acid 200 *μ*mol/L group; (e): Ghrelin 100 nmol/L+ palmitic acid 200 *μ*mol/L group. Values are expressed as mean ± SEM (*P < 0.05 versus control; ^#^ *P* < 0.05 versus palmitic acid 200 *μ*mol/L group).

**Figure 5:**
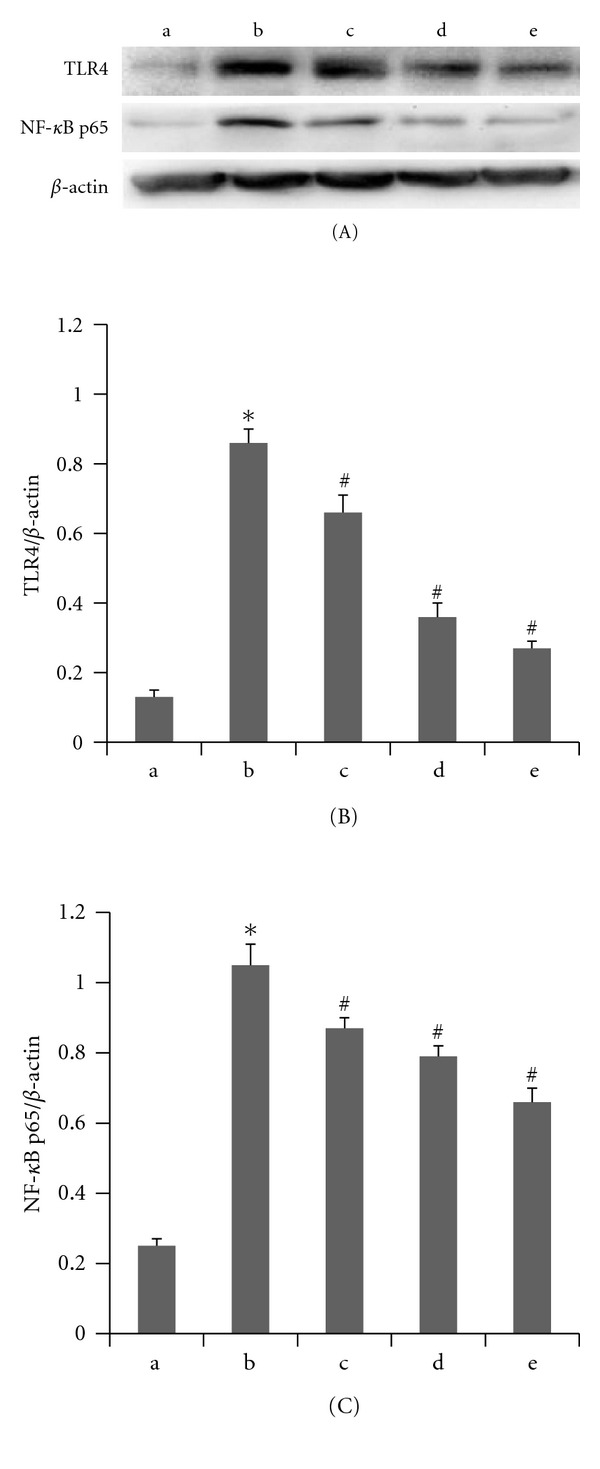
Acylated ghrelin pretreatment decreased palmitic acid-induced level of TLR4 protein and NF-*κ*B p65 phosphorylationprotein in THP-1 macrophages. (a) control; (b) palmitic acid 200 *μ*mol/L group; (c) ghrelin 1 nmol/L+ palmitic acid 200 *μ*mol/L group; (d) ghrelin 10 nmol/L+ palmitic acid 200 *μ*mol/L group; (e) ghrelin 100 nmol/L+ palmitic acid 200 *μ*mol/L group. (A) Western blotting electropherogram of TLR4 (96 KD), NF-*κ*B p65 (65 KD), and *β*-actin (42 KD). (B) Acylated ghrelin pretreatment decreased palmitic acid-induced level of TLR4 protein in a dose-dependent in THP-1 macrophages, with values expressed as mean ± SEM (*P < 0.05 versus control; ^#^ *P* < 0.05 versus palmitic acid 200 *μ*mol/L group). (C) Acylated ghrelin pretreatment decreased palmitic acid-induced level of NF-*κ*B p65 phosphorylationprotein in a dose-dependent in THP-1 macrophages, with values expressed as mean ± SEM (*P < 0.05 versus control; ^#^ *P* < 0.05 versus palmitic acid 200 *μ*mol/L group).

**Figure 6:**
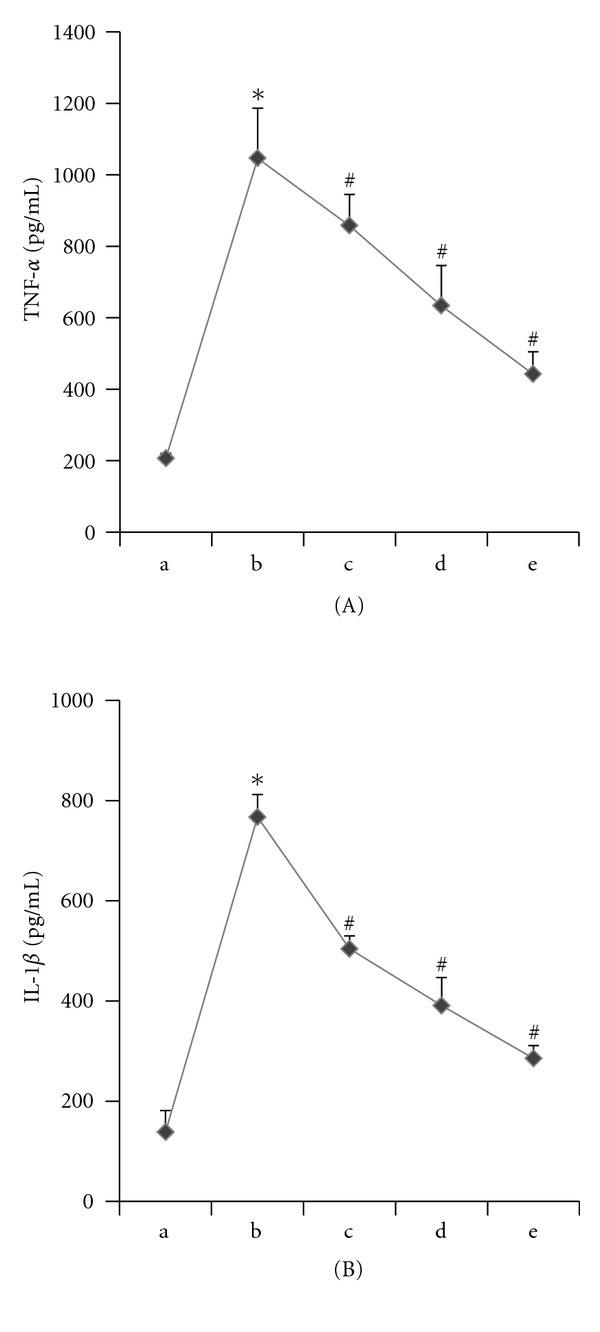
Acylated ghrelin pretreatment inhibited palmitic acid-induced release of IL-1*β* and TNF-*α* in THP-1 macrophages. (a) control; (b) palmitic acid 200 *μ*mol/L group; (c) ghrelin 1 nmol/L+ palmitic acid 200 *μ*mol/L group; (d) ghrelin 10 nmol/L+ palmitic acid 200*μ*mol/L group; (e) ghrelin 100 nmol/L+ palmitic acid 200 *μ*mol/L group. (A) Acylated ghrelin pretreatment inhibited palmitic acid-induced release of TNF-*α* in a dose-dependent in THP-1 macrophages, with values expressed as mean ± SEM (*P < 0.05 versus control; ^#^ *P* < 0.05 versus palmitic acid 200 *μ*mol/L group). (B) Acylated ghrelin pretreatment inhibitedpalmitic acid-induced release of IL-1*β* in a dose-dependent in THP-1 macrophages, with values expressed as mean ± SEM (*P < 0.05 versus control; ^#^ *P* < 0.05 versus palmitic acid 200 *μ*mol/L group).

We incubated THP-1 macrophages in different concentrations of octanoylated ghrelin (0 nmol/L, 1 nmol/L, 10 nmol/L, and 100 nmol/L) for 4 hours. Palmitic acid (200 *μ*mol/L) was subsequently added prior to additional 12 hours of incubation. The RT-PCR results showed that relative to the 200 *μ*mol/L palmitic acid-incubated group, the addition of octanoylated ghrelin caused a decrease in the TLR4 mRNA expression in a dose-dependent manner (*P* < 0.05) (Figure 4). The western blot analyses showed that octanoylated ghrelin amendment led to decreases in TLR4 protein levels and NF-*κ*B p65 phosphorylation in THP-1 macrophages in a dose-dependent manner (*P* < 0.05) relative to the 200 *μ*mol/L palmitic acid-incubated group (Figure 5). The ELISA results showed that octanoylated ghrelin caused a decrease in the secretion of IL-1*β* and TNF-*α* by THP-1 macrophages in a dose-dependent manner (*P* < 0.05) relative to the 200 *μ*mol/L palmitic acid-incubated group (Figure 6).

## 4. Discussion

******We chose to incubate THP-1 macrophages with palmitic acid, which is a type of saturated fatty acid. The results showed that the concentrations of IL-1*β* and TNF-*α* in the supernatants of the cultured cells significantly increased with increasing palmitic acid concentrations. This observation suggested that palmitic acid could have a proinflammatory effect in a dose-dependent manner. 

TLR4 is a member of the TLRs that have a natural pattern recognition. The function of TLR4 is to mediate trans-membrane signaling transduction in which TLR4 could serve as a bridge that links innate immunity, lipid metabolism, insulin resistance, and vascular inflammation. TLR4 widely recognizes specific pathogen-associated molecular patterns, such as the LPS layer of gram-negative bacilli and FFAs that are secreted by adipocytes, and couples signal transduction pathways to activate innate immune cells and inflammatory cells, which results in a series of immune and inflammatory responses and leads to the synthesis and release of cytokines and inflammatory mediators. TLR4 induces a high level of expression of target genes with proinflammatory abilities by regulating the activity of signaling-dependent transcription factors (such as NF-*κ*B). The NF-*κ*B pathway plays an important role in TLR4-mediated immune regulation [23]. In TLR4-deficient mice, this vascular proinflammatory gene cannot be expressed, regardless of the extent of obesity, dyslipidemia, or high fat intake [24]. Increased plasma levels of palmitic acid can serve as the natural endogenous ligand of TLR4 that results in an abnormal TLR4 gene expression and TLR/NF-*κ*B signal transduction and increases in the gene expression of the inflammatory cytokine IL-6 and SOD2 (mitochondrial superoxide dismutase), which could be one of the pathological mechanisms of insulin resistance [25]. Our study confirmed that palmitic acid could induce the activation of the TLR4/NF-*κ*B signaling pathway in THP-1 macrophages in a dose-dependent manner, thereby establishing a cellular model of metabolic inflammation.

Ghrelin is the endogenous ligand of GHSR and is a 28-amino acid peptide. The two forms of ghrelin that are found in the body are n-octanoyl ghrelin, which is octanoylated with an n-octanoyl group on Ser3, and des-acyl ghrelin. The former plays important roles in biological activities. The octanoylated form is the ligand of GHSR-1 and is able to pass through the blood-brain barrier to bind with GHSR-1*α* in the central nervous system to exert its endocrine function. Ghrelin is mainly secreted by gastric X/A-like endocrine cells. A small amount of ghrelin secretion is also observed in the intestines, pancreas, kidneys, immune system, placenta, testis, pituitary, and hypothalamus. The receptor of ghrelin, GHSR, is a G protein-coupled receptor that exists as two types, that is, GHSR-1a and GHSR-1b. Most of the physiological effects of ghrelin are mediated by GHSR-1a. GHSR-1a mRNA expression mainly occurs in the hypothalamus and pituitary gland but also occurs in the peripheral tissues, such as the gastrointestinal tract, thyroid, pancreas, myocardium, and kidneys [26, 27]. After binding with its receptors, octanoylated ghrelin has many biological effects, including the promotion of the release of growth hormone, increasing food intake, the regulation of lipid metabolism, and the inhibition of inflammatory cytokine secretion. Ghrelin can relax blood vessels to lower blood pressure, reduce myocyte apoptosis, protect endothelial cells, improve weakened cardiac function, and inhibit the stress reaction of the endoplasmic reticulum; ghrelin also potentially has protective effects on the cardiovascular system [28, 29]. The levels of plasma ghrelin are negatively correlated with body weight, fasting insulin levels, and insulin resistance (HOMA-IR) and are positively correlated with the insulin sensitivity index (ISI). These relationships suggest that the reduced ghrelin levels in patients with obesity may lead to insulin resistance. However, the mechanism causing the negative correlation between plasma ghrelin and insulin resistance has not been elucidated [30, 31]. 

Ghrelin plays an important role in the inflammatory response, such as the inhibition of the release of inflammatory cytokines. A recent study [18] suggested that ghrelin showed strong anti-inflammatory effects by regulating the secretion of macrophage proinflammatory cytokines, such as IL-1*β* and TNF-*α*, and the anti-inflammatory cytokine IL-10 after LPS stimulation through the NF-*κ*B and P38 mitogen-activated protein kinase (MAPK) signaling pathways. In lymphocytes and monocytes, ghrelin can also specifically inhibit the synthesis of leptin and LPS-induced proinflammatory cytokines, such as IL-1, IL-6, and TNF-*α* [19]. An *in vitro* experiment showed that ghrelin could inhibit the secretion of proinflammatory cytokines from human endothelial cells, the adhesion of monocytes, and the activity of NF-*κ*B. The intravenous administration of ghrelin can also inhibit the *in vivo* synthesis of endotoxin-induced proinflammatory cytokines in rats [20]. Recently, an additional study showed that ghrelin could increase NF-*κ*B activity in B-lymphocytes [32]. Our previous study showed that the plasma from obese patients could promote the release of inflammatory factors through activation of the TLR4/NF-*κ*B pathway [33]. Newgard et al. [34] found that the ghrelin levels of obese patients were significantly lower than those of control individuals (*P* < 0.0001). Our study demonstrated that ghrelin could inhibit the release of inflammatory cytokines via the inhibition of palmitic acid-induced activation of the TLR4/NF-*κ*B signaling pathway in THP-1 macrophages in a dose-dependent manner. 

The activation of the TLR4/NF-*κ*B signaling pathway can induce and aggravate insulin resistance, which leads to obesity and related metabolic disorders. Ghrelin can have an anti-inflammatory effect by inhibiting the TLR4/NF-*κ*B pathway's activity. Therefore, The anti-inflammatory effect of ghrelin might be used to explore new drugs to prevent and therapy obesity, diabetes, and other metabolic inflammatory diseases.

## References

[1] Hotamisligil GS, Shargill NS, Spiegelman BM (1993). Adipose expression of tumor necrosis factor-*α*: direct role in obesity-linked insulin resistance. *Science*.

[2] Ghanim H, Aljada A, Hofmeyer D, Syed T, Mohanty P, Dandona P (2004). Circulating mononuclear cells in the obese are in a proinflammatory state. *Circulation*.

[3] Wu H, Ghosh S, Perrard XD (2007). T-cell accumulation and regulated on activation, normal T cell expressed and secreted upregulation in adipose tissue in obesity. *Circulation*.

[4] Olefsky JM, Glass CK (2009). Macrophages, inflammation, and insulin resistance. *Annual Review of Physiology*.

[5] Kim F, Tysseling KA, Rice J (2005). Free fatty acid impairment of nitric oxide production in endothelial cells is mediated by IKK*β*. *Arteriosclerosis, Thrombosis, and Vascular Biology*.

[6] Roden M, Price TB, Perseghin G (1996). Mechanism of free fatty acid-induced insulin resistance in humans. *Journal of Clinical Investigation*.

[7] Shoelson SE, Lee J, Yuan M (2003). Inflammation and the IKK*β*/I*κ*B/NF-*κ*B axis in obesity- and diet-induced insulin resistance. *International Journal of Obesity*.

[8] Hotamisligil GS (2003). Inflammatory pathways and insulin action. *International Journal of Obesity*.

[9] Cai D, Yuan M, Frantz DF (2005). Local and systemic insulin resistance resulting from hepatic activation of IKK-*β* and NF-*κ*B. *Nature Medicine*.

[10] Shoelson SE, Lee J, Goldfine AB (2006). Inflammation and insulin resistance. *Journal of Clinical Investigation*.

[11] Kim F, Pham M, Luttrell I (2007). Toll-like receptor-4 mediates vascular inflammation and insulin resistance in diet-induced obesity. *Circulation Research*.

[12] Shi H, Kokoeva MV, Inouye K, Tzameli I, Yin H, Flier JS (2006). TLR4 links innate immunity and fatty acid-induced insulin resistance. *Journal of Clinical Investigation*.

[13] Akira S, Takeda K (2004). Toll-like receptor signalling. *Nature Reviews Immunology*.

[14] Lee JY, Sohn KH, Rhee SH, Hwang D (2001). Saturated fatty acids, but not unsaturated fatty acids, induce the expression of cyclooxygenase-2 mediated through toll-like receptor 4. *Journal of Biological Chemistry*.

[15] Suganami T, Tanimoto-Koyama K, Nishida J (2007). Role of the Toll-like receptor 4/NF-*κ*B pathway in saturated fatty acid-induced inflammatory changes in the interaction between adipocytes and macrophages. *Arteriosclerosis, Thrombosis, and Vascular Biology*.

[16] Schäffler A, Schölmerich J (2010). Innate immunity and adipose tissue biology. *Trends in Immunology*.

[17] Waseem T, Duxbury M, Ito H, Ashley SW, Robinson MK (2008). Exogenous ghrelin modulates release of pro-inflammatory and anti-inflammatory cytokines in LPS-stimulated macrophages through distinct signaling pathways. *Surgery*.

[18] Dixit VD, Schaffer EM, Pyle RS (2004). Ghrelin inhibits leptin- and activation-induced proinflammatory cytokine expression by human monocytes and T cells. *Journal of Clinical Investigation*.

[19] Li WG, Gavrila D, Liu X (2004). Ghrelin inhibits proinflammatory responses and nuclear factor-*κ*B activation in human endothelial cells. *Circulation*.

[20] Kheradmand A, Alirezaei M, Birjandi M (2010). Ghrelin promotes antioxidant enzyme activity and reduces lipid peroxidation in the rat ovary. *Regulatory Peptides*.

[21] Liu L, Xu H, Jiang H, Wang J, Song N, Xie J (2010). Ghrelin prevents 1-methyl-4-phenylpyridinium ion-induced cytotoxicity through antioxidation and NF-*κ*B modulation in MES23.5 cells. *Experimental Neurology*.

[22] Imazu Y, Yanagi S, Miyoshi K (2011). Ghrelin ameliorates bleomycin-induced acute lung injury by protecting alveolar epithelial cells and suppressing lung inflammation. *European Journal of Pharmacology*.

[23] Akira S, Takeda K (2004). Toll-like receptor signalling. *Nature Reviews Immunology*.

[24] Tsukumo DML, Carvalho-Filho MA, Carvalheira JBC (2007). Loss-of-function mutation in toll-like receptor 4 prevents diet-induced obesity and insulin resistance. *Diabetes*.

[25] Reyna SM, Ghosh S, Tantiwong P (2008). Elevated toll-like receptor 4 expression and signaling in muscle from insulin-resistant subjects. *Diabetes*.

[26] Broglio F, Gottero C, Arvat E, Ghigo E (2003). Endocrine and non-endocrine actions of ghrelin. *Hormone Research*.

[27] Soares JB, Leite-Moreira AF (2008). Ghrelin, des-acyl ghrelin and obestatin: three pieces of the same puzzle. *Peptides*.

[28] Moazed B, Quest D, Gopalakrishnan V (2009). Des-acyl ghrelin fragments evoke endothelium-dependent vasodilatation of rat mesenteric vascular bed via activation of potassium channels. *European Journal of Pharmacology*.

[29] Zhang GG, Teng X, Liu Y (2009). Inhibition of endoplasm reticulum stress by ghrelin protects against ischemia/reperfusion injury in rat heart. *Peptides*.

[30] Pöykkö SM, Kellokoski E, Hörkkö S, Kauma H, Kesäniemi YA, Ukkola O (2003). Low plasma ghrelin is associated with insulin resistance, hypertension, and the prevalence of type 2 diabetes. *Diabetes*.

[31] Ukkola O, Pöykkö SM, Antero Kesäniemi Y (2006). Low plasma ghrelin concentration is an indicator of the metabolic syndrome. *Annals of Medicine*.

[32] Sung EZH, Da Silva NF, Goodyear SJ, McTernan PG, Arasaradnam RP, Nwokolo CU (2011). Ghrelin promotes nuclear factor *κ*-B activation in a human B-lymphocyte cell line. *Molecular Biology Reports*.

[33] Yao L, Xiao Y, Liu SP, Xu AM, Zhou ZG (2010). Serum of obesity induce the activation of TLR4/NF-*κ*B signaling pathway on THP-1 cell line. *Zhonghua Yi Xue Za Zhi*.

[34] Newgard CB, An J, Bain JR (2009). A branched-chain amino acid-related metabolic signature that differentiates obese and lean humans and contributes to insulin resistance. *Cell Metabolism*.

